# Supplementary material for: Atomically defined angstrom-scale all-carbon junctions
Source: Nat Commun. 2019 Apr 15;10:1748. doi: 10.1038/s41467-019-09793-8 (PMC6465289; doi:10.1038/s41467-019-09793-8)
Supplement: Supplementary file 1 — Supplementary Information [file 41467_2019_9793_MOESM1_ESM.pdf]

Supplementary Information for

Atomically defined angstrom-scale all-carbon junctions

Tan et al.

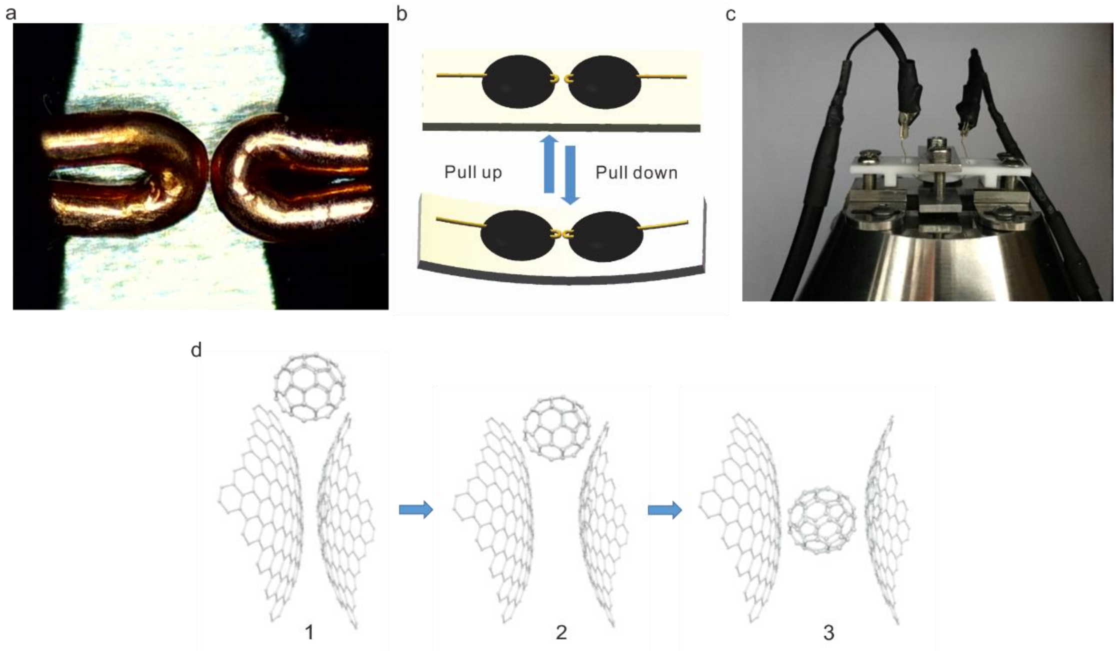

Supplementary Figure 1 | a, b, The photo and schematic of graphene chip. **c,** The photo of our home-made MCBJ experiment setup. **d,** The schematic of the formation of graphene/fullerene/graphene junction.

Supplementary Note 1.

Discussion for Supplementary Figure 1d:

Supplementary Figure 1d gives a schematic of the evolution of a graphene/fullerene/graphene junction. Panels 1, 2 and 3 present the state in which the electrodes are about to be opened, part of the electrodes are opened, and the electrodes are fully opened, respectively. Fullerenes were assembled on graphene by π-π interaction before the electrodes were opened. In this way, fullerenes provide a pathway for charge transport between electrodes at the moment the electrodes are opened, and the conductance of the whole molecular junction has been detected, and then the molecular junction gradually goes through configurations 2, 3. Due to the decrease of through-space tunneling and the reduced fullerene-graphene coupling, the conductance corresponding to

the configurations 2, 3 shown in the figure may vary slightly with the increasing distance between the two graphene electrodes.

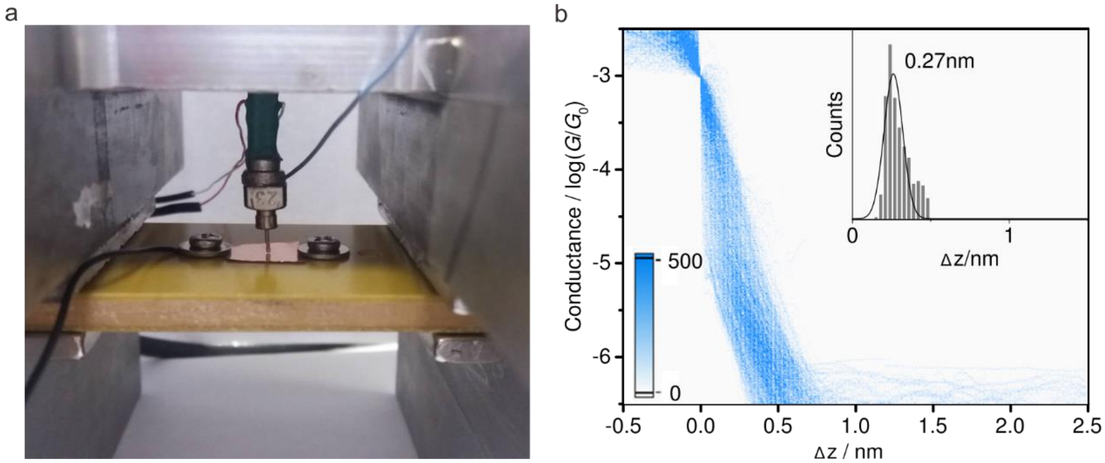

Supplementary Figure 2 | a, The photo of graphene STM-BJ experiment setup. **b,** 2D conductance histogram of pure decane using graphene STM-BJ technique, the top right insert is the relative displacement distribution from 1.0×10^{-4} to 1.0×10^{-6} G_0.

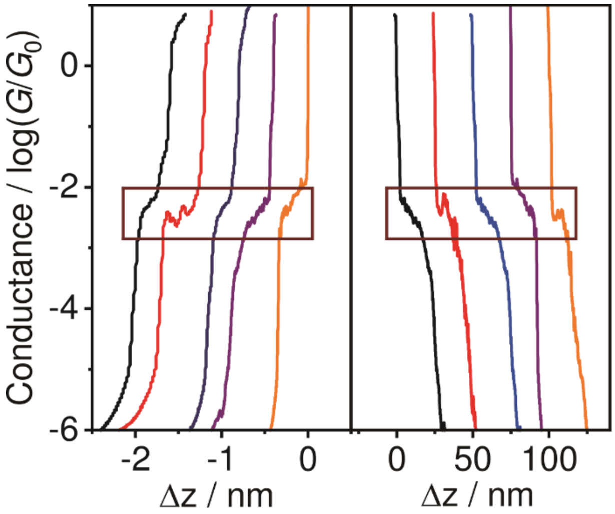

Supplementary Figure 3 | The individual conductance-displacement curves of a control experiment of graphene electrodes with the high set trigger at 10 G_0 (The left panel is the closing process and the right panel is the opening process).

Supplementary Note 2.

Discussion for Supplementary Figure 3:

As shown in Supplementary Figure 3, most of the closing and opening curves show a similar conductance feature between 10^{-2} G_0 and 10^{-3} G_0 (marked in the dark red square), which corresponds to the state in which the two graphene electrodes are contacting each other. Meanwhile, the opening traces show the relatively long distance to achieve hard contact. It should also be noticed that the displacement to break the graphene-graphene contact is tens of nanometers, which is significantly larger than the π-π interaction distance (usually considered to be about 0.35 nanometers), suggesting the deformation of the graphene layers from the copper surface may happen with hard contact mode. After 10^{-3} G_0, the direct tunneling curves demonstrate that the electrodes are separated and the current decreases exponentially with the increase of distance. Due to the random contact angle and the deformation of the two graphene electrodes, the point contact conductance of graphene varies a lot from sample to sample, but the set higher trigger of 3.2×10^{-2} G_0 is below the determined conductance range of the contracted graphene electrodes. The data also show that the range from 3.2×10^{-2} G_0 to 1.0×10^{-6} G_0 provides a wide enough window to measure the conductance of graphene/fullerene/graphene junctions.

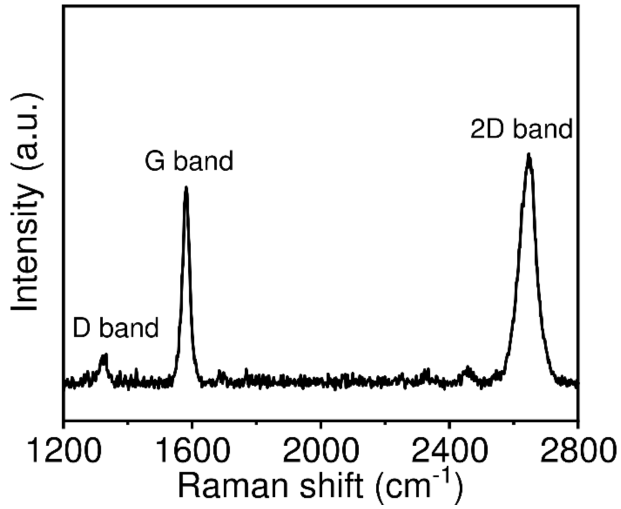

Supplementary Figure 4 | Raman characterization of CVD graphene on copper wire.

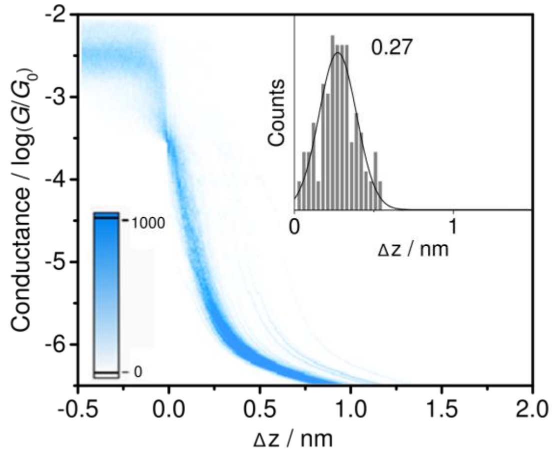

Supplementary Figure 5 | The 2D conductance-displacement histogram of decane，the top right insert is the relative displacement distribution ranging from 1.0 $\times 10^{-4}$ to 1.0 $\times 10^{-6}$ G_0.

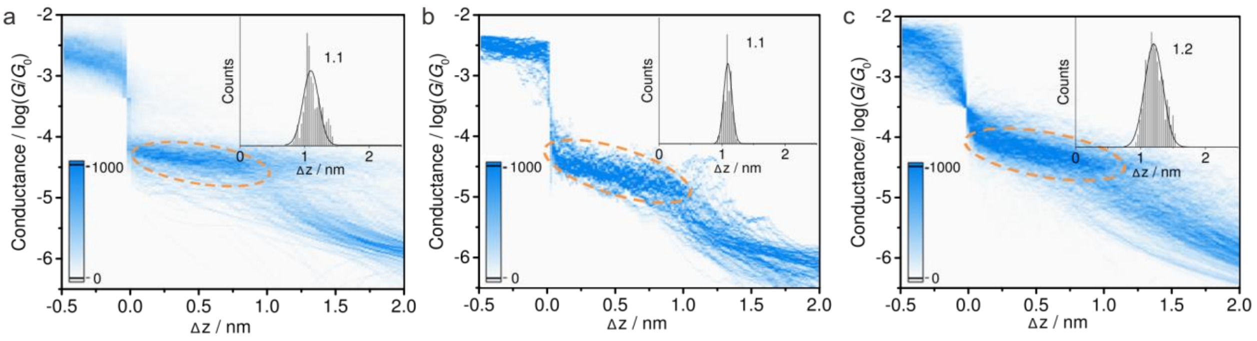

Supplementary Figure 6 | 2D conductance histogram of graphene/single-C_{70}, C_{76}, C_{90}/graphene junctions obtained from around 1000 traces. The top right insert is the relative displacement distribution, from a to c, the statistics range from 3.2 $\times 10^{-4}$ to 1.0 $\times 10^{-5}$ G_0, 6.3 $\times 10^{-6}$ G_0, 1.6 $\times 10^{-5}$ G_0 respectively.

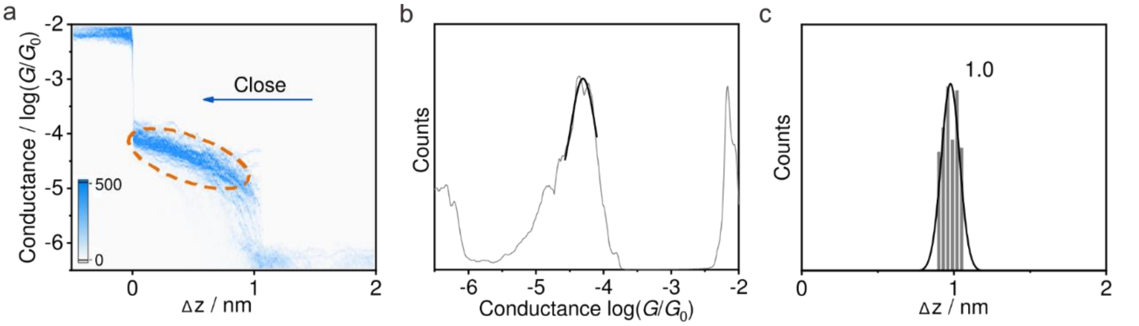

Supplementary Figure 7 | Data of the closing process of graphene/single-C_{60}/graphene junctions. a, b, 2D and 1D conductance histogram of the closing processes of graphene/single-C_{60}/graphene junctions. **c,** The relative displacement distribution determined from 3.2×10^{-4} to 4.0×10^{-6} G_0.

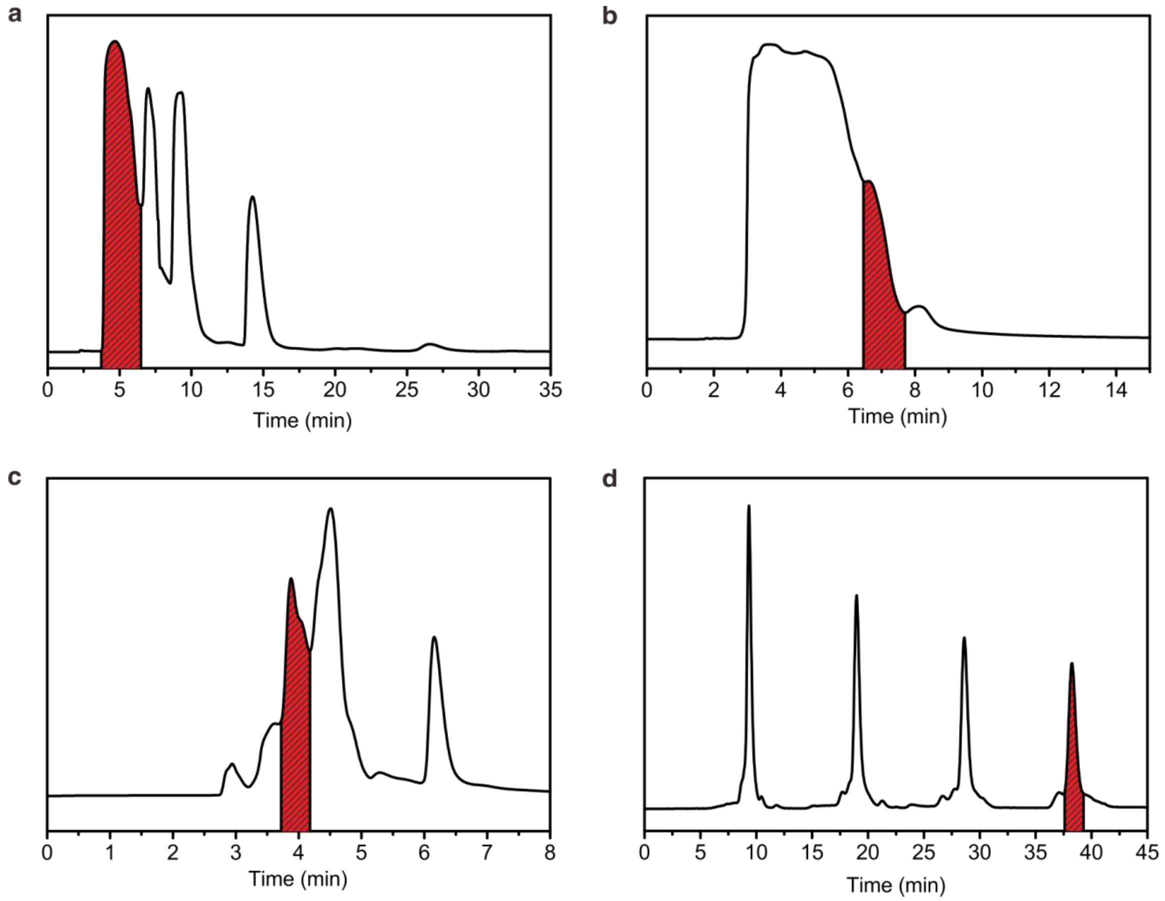

Supplementary Figure 8 | Multi-stage HPLC separation of $C_{50}H_{10}$. a, Column: buckyprep column (20mm × 250 mm), flow rate: 20 mL min^{-1}, eluent: toluene. **b,** Column: 5PBB column (10mm × 250 mm), flow rate: 4 mL min^{-1}, eluent: toluene. **c,** Column: buckyprep column (10mm × 250 mm), flow rate: 4 mL min^{-1}, eluent: toluene. **d,** Column: 5PBB column (10mm × 250 mm), flow rate: 4 mL min^{-1}, eluent: toluene. The regions of collected components are highlighted as red shadow.

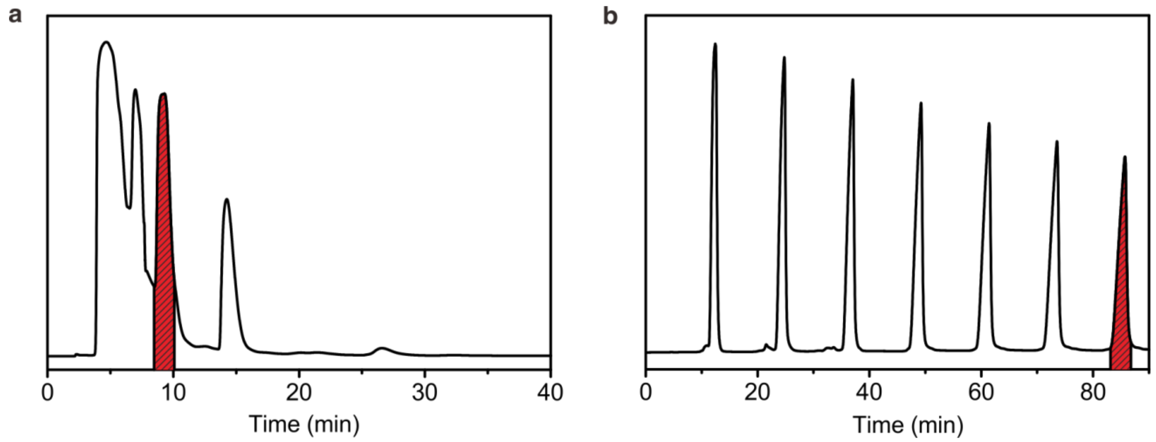

Supplementary Figure 9 | Multi-stage HPLC separation of C_{60}. a, Column: buckyprep column (20mm × 250 mm), flow rate: 20 mL min^{-1}, eluent: toluene. **b,** Column: 5PBB column (10mm × 250 mm), flow rate: 4 mL min^{-1}, eluent: toluene. The regions of collected components are highlighted as red shadow.

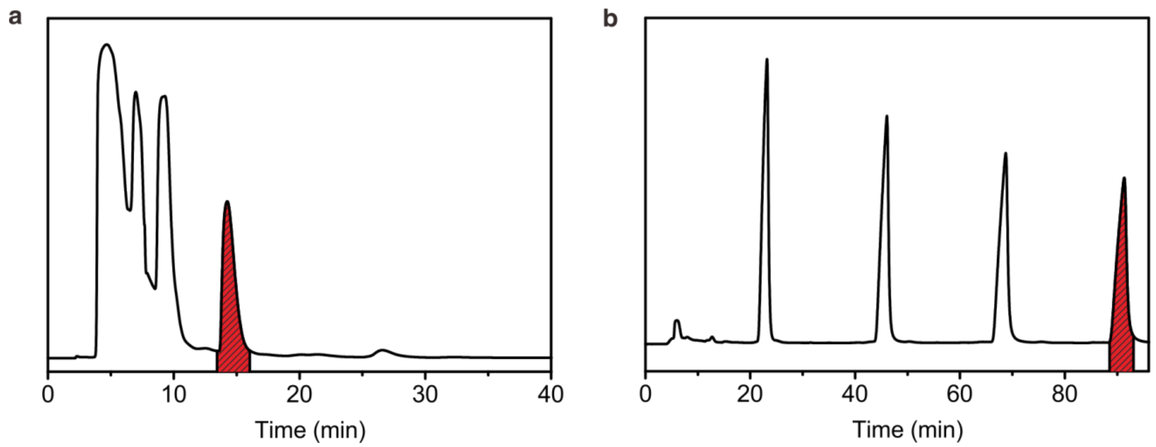

Supplementary Figure 10 | Multi-stage HPLC separation of C_{70}. a, Column: buckyprep column (20mm × 250 mm), flow rate: 20 mL min^{-1}, eluent: toluene. **b,** Column: 5PBB column (10mm × 250 mm), flow rate: 4 mL min^{-1}, eluent: toluene. The regions of collected components are highlighted as red shadow.

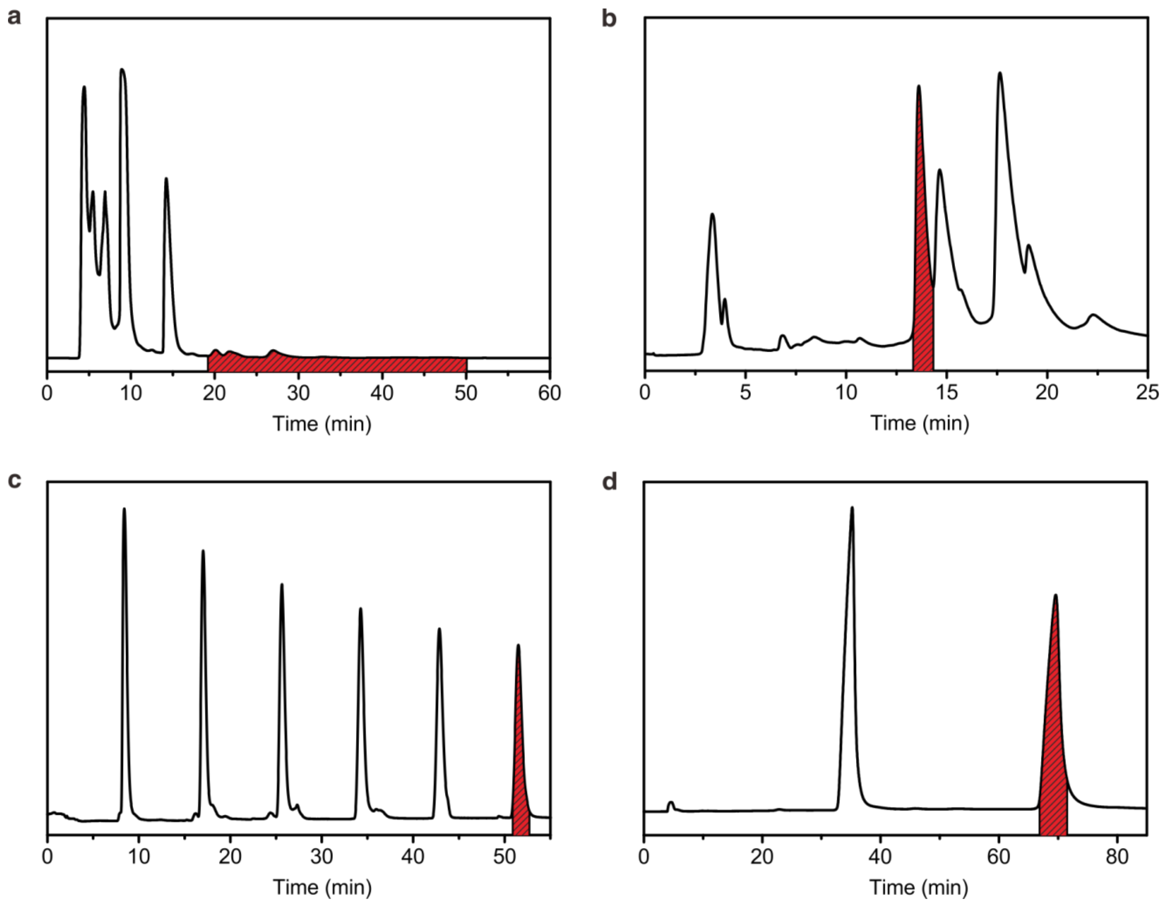

Supplementary Figure 11 | Multi-stage HPLC separation of C_{76}. a, Column: buckyprep column (20mm×250 mm), flow rate: 20 mL min^{-1}, eluent: toluene. **b,** Column: 5PBB column (10mm×250 mm), flow rate: 8 mL min^{-1}, eluent: toluene. **c,** Column: 5NPE column (10mm×250 mm), flow rate: 4 mL min^{-1}, eluent: toluene. **d,** Column: 5PBB column (10mm×250 mm), flow rate: 4 mL min^{-1}, eluent: toluene. The regions of collected components are highlighted as red shadow.

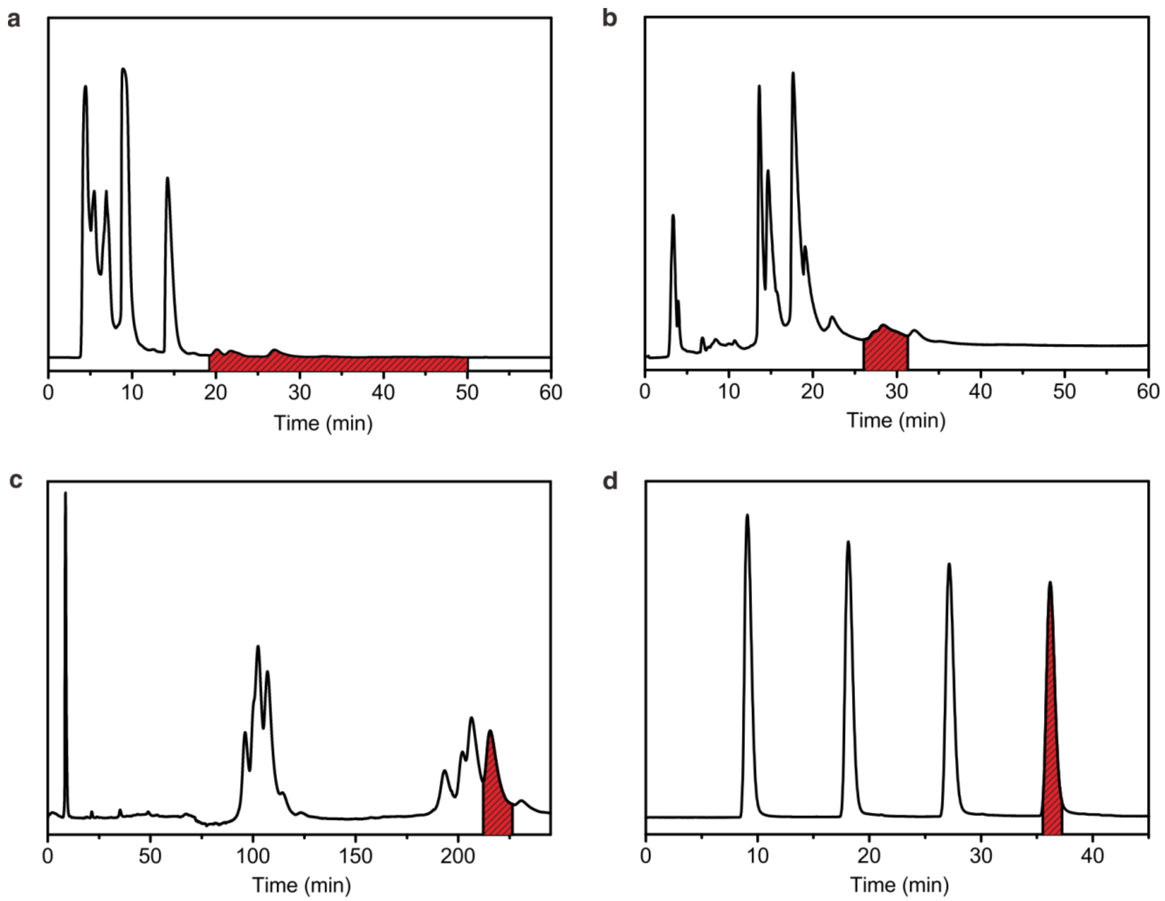

Supplementary Figure 12 | Multi-stage HPLC separation of C_{90}. a, Column: buckyprep column (20mm × 250 mm), flow rate: 20 mL min^{-1}, eluent: toluene. **b,** Column: 5PBB column (10mm × 250 mm), flow rate: 8 mL min^{-1}, eluent: toluene. **c,** Column: buckyprep column (20mm × 250 mm), flow rate: 8 mL min^{-1}, eluent: toluene. **d,** Column: 5NPE column (10mm × 250 mm), flow rate: 4 mL min^{-1}, eluent: toluene. The regions of collected components are highlighted as red shadow.

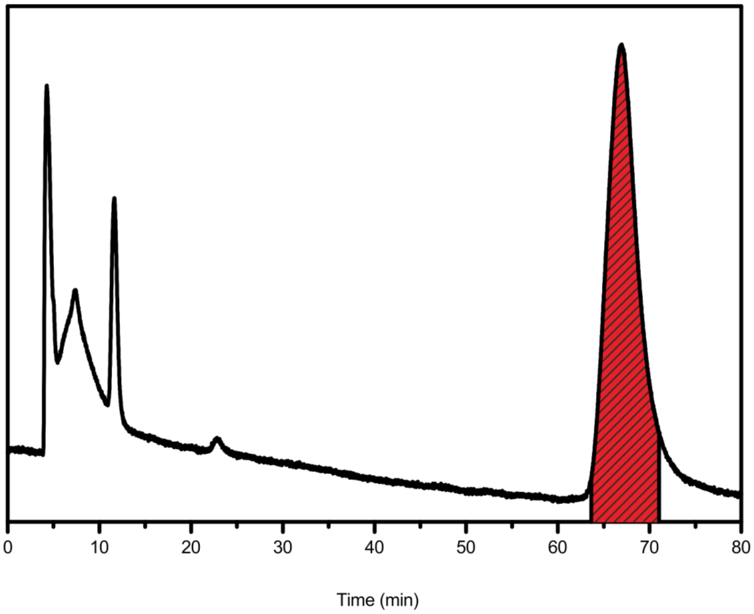

Supplementary Figure 13 | HPLC separation of C_{120}. Column: 5PBB column (10mm ×250 mm), flow rate: 4 mL min^{-1}, eluent: toluene. The region of collected component is highlighted as red shadow.

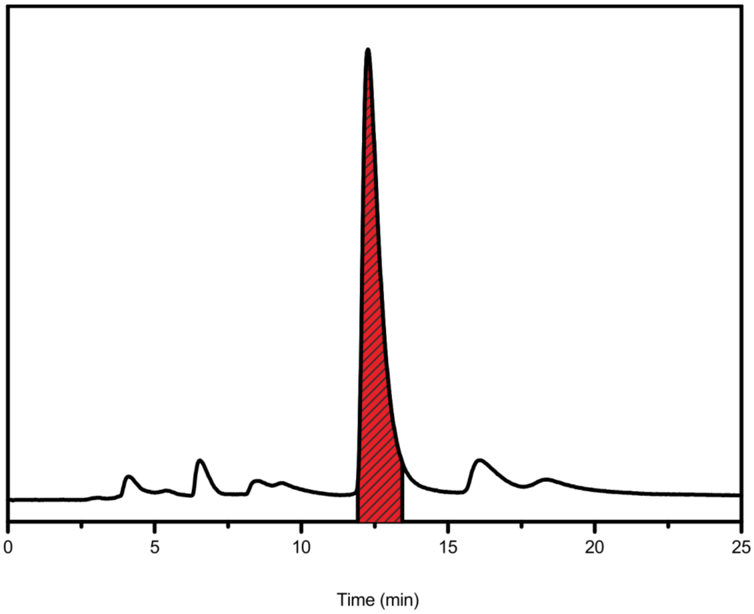

Supplementary Figure 14 | HPLC separation of $(C_{59}N)_2$. Column: buckyprep column (10mm×250 mm), flow rate: 4 mL min^{-1}, eluent: toluene. The region of collected component is highlighted as red shadow.

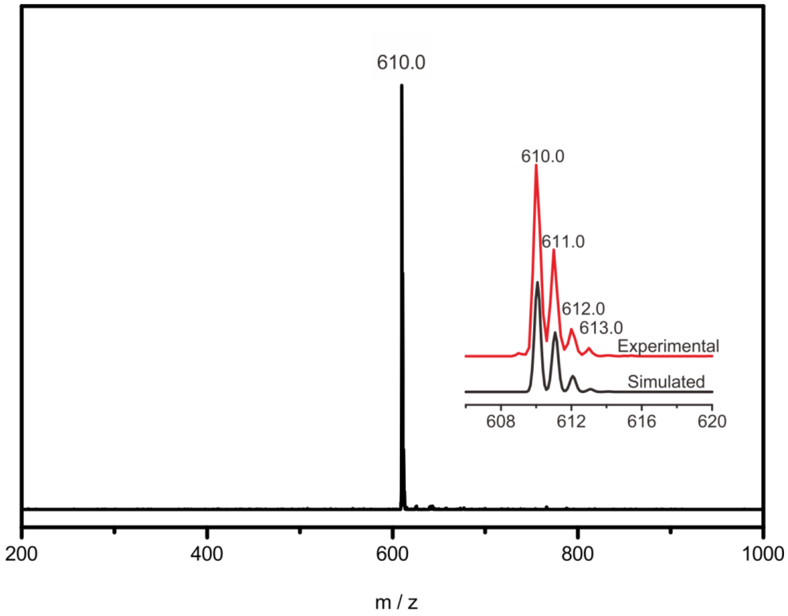

Supplementary Figure 15 | The APCI-MS of the $C_{50}H_{10}$ with experimental and simulated isotopic distribution inset. The mass spectrum of the purified sample of $C_{50}H_{10}$ shows a molecular ion peak of 610.0 m/z, in agreement with the chemical composition of $C_{50}H_{10}$.

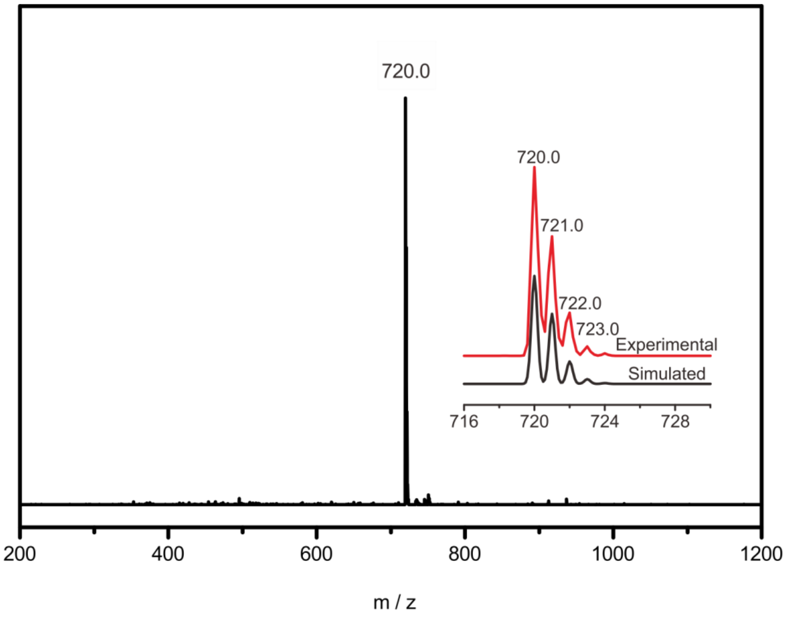

Supplementary Figure 16 | The APCI-MS of the C_{60} with experimental and simulated isotopic distribution inset. The mass spectrum of the purified sample of C_{60} shows a molecular ion peak of 720.0 m/z, in agreement with the chemical composition of C_{60}.

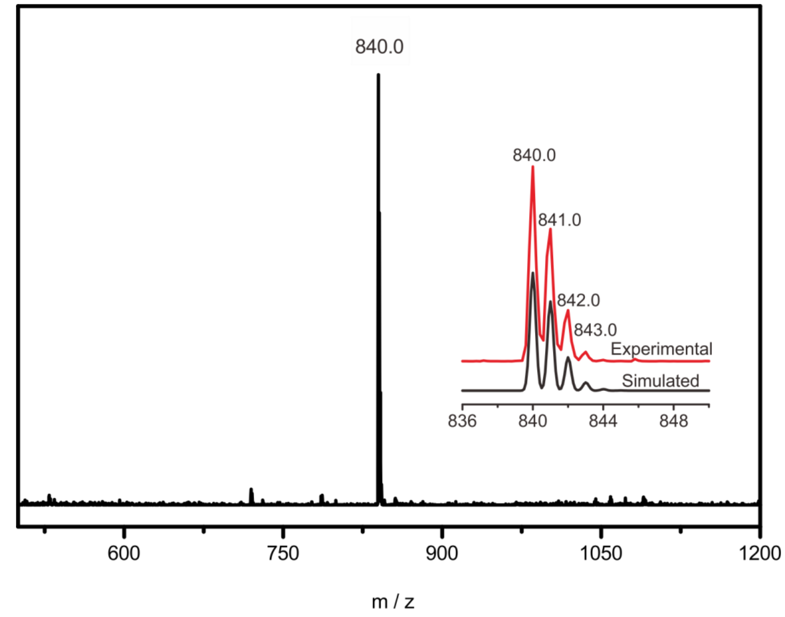

Supplementary Figure 17 | The APCI-MS of the C_{70} with experimental and simulated isotopic distribution inset. The mass spectrum of the purified sample of C_{70} shows a molecular ion peak of 840.0 m/z, in agreement with the chemical composition of C_{70}.

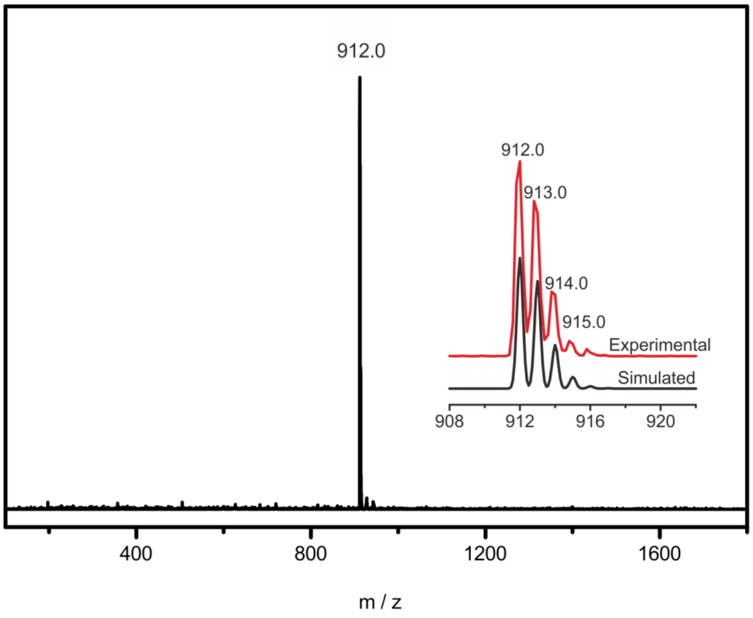

Supplementary Figure 18 | The APCI-MS of the C_{76} with experimental and simulated isotopic distribution inset. The mass spectrum of the purified sample of C_{76} shows a molecular ion peak of 912.0 m/z, in agreement with the chemical composition of C_{76}.

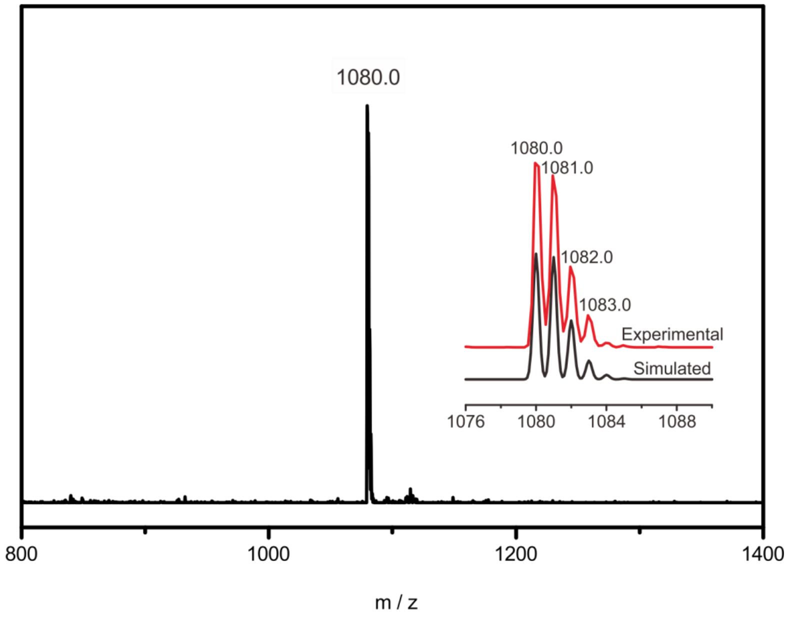

Supplementary Figure 19 | The APCI-MS of the C_{90} with experimental and simulated isotopic distribution inset. The mass spectrum of the purified sample of C_{90} shows a molecular ion peak of 1080.0 m/z, in agreement with the chemical composition of C_{90}.

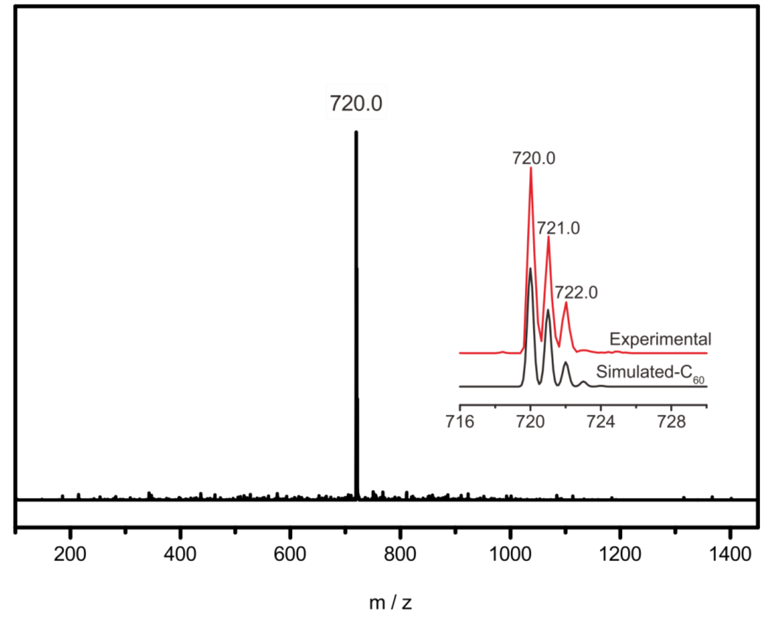

Supplementary Figure 20 | The APCI-MS of the C_{120} with experimental and simulated C_{60} isotopic distribution inset.

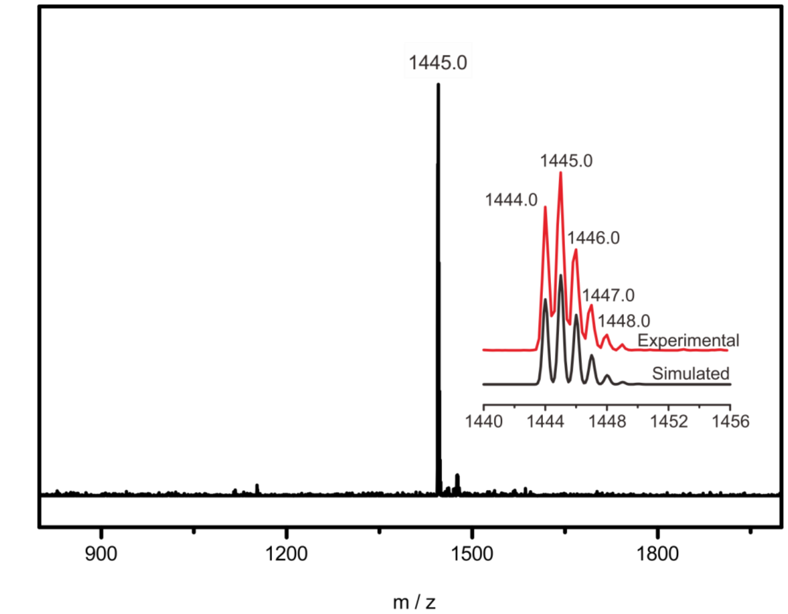

Supplementary Figure 21 | The APCI-MS of the $(C_{59}N)_2$ with experimental and simulated isotopic distribution inset.

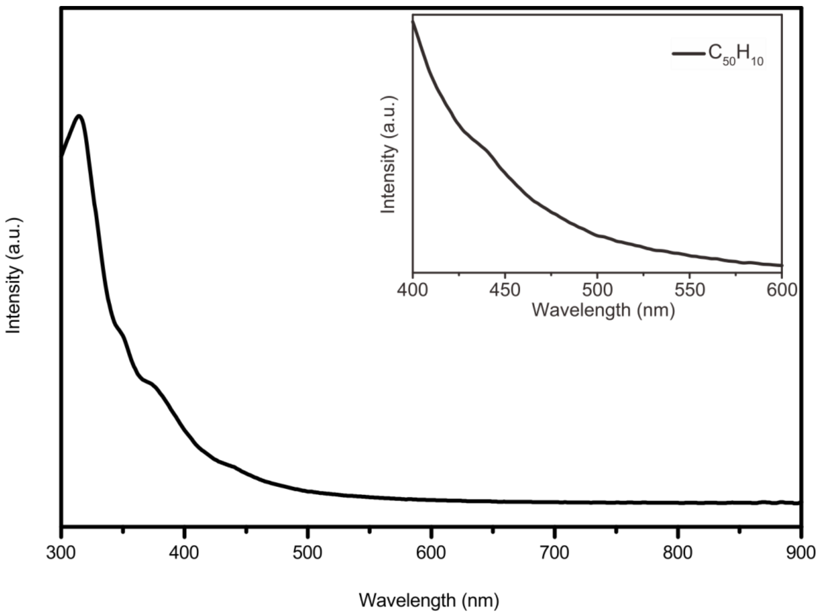

Supplementary Figure 22 | UV-Vis spectrum of $C_{50}H_{10}$ in cyclohexane solution. Enlarged absorption spectra in the region from 400 to 600 nm inset.

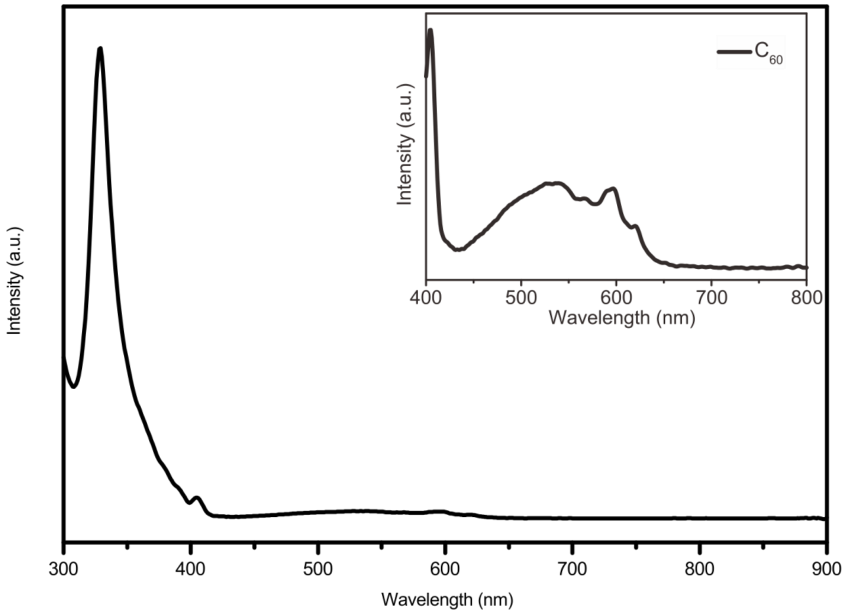

Supplementary Figure 23 | UV-Vis spectrum of C_{60} in n-hexane solution. Enlarged absorption spectra in the region from 400 to 800 nm inset.

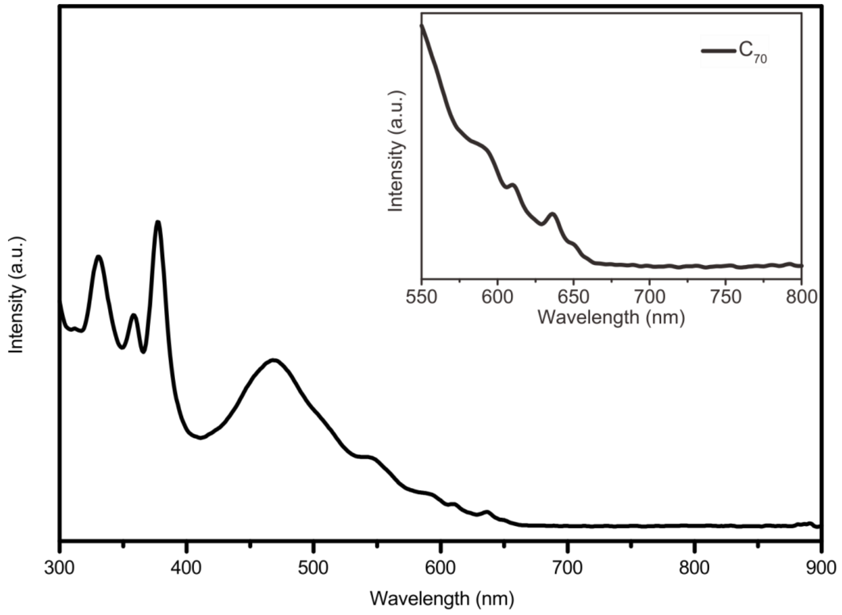

Supplementary Figure 24 | UV-Vis spectrum of C_{70} in n-hexane solution. Enlarged absorption spectra in the region from 550 to 800 nm inset.

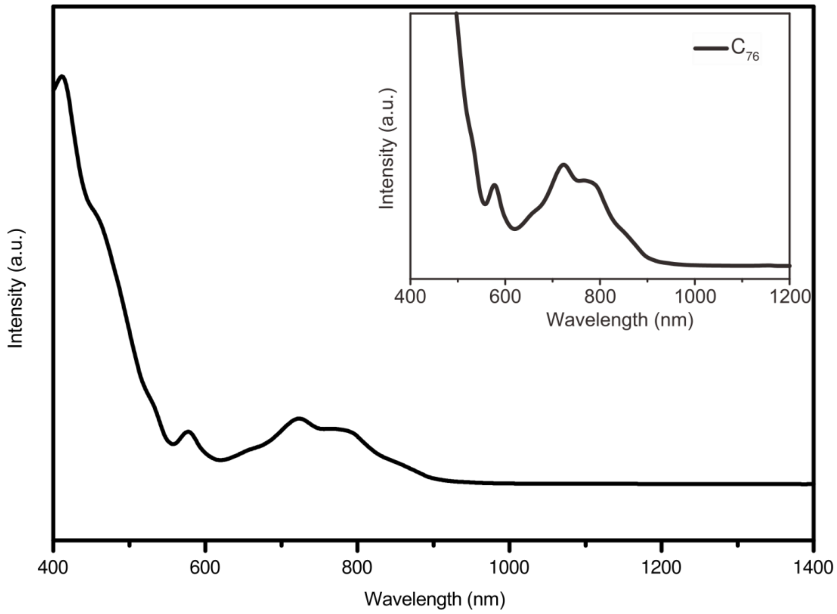

Supplementary Figure 25 | UV-Vis spectrum of C_{76} in carbon disulfide solution. Enlarged absorption spectra in the region from 400 to 1200 nm inset.

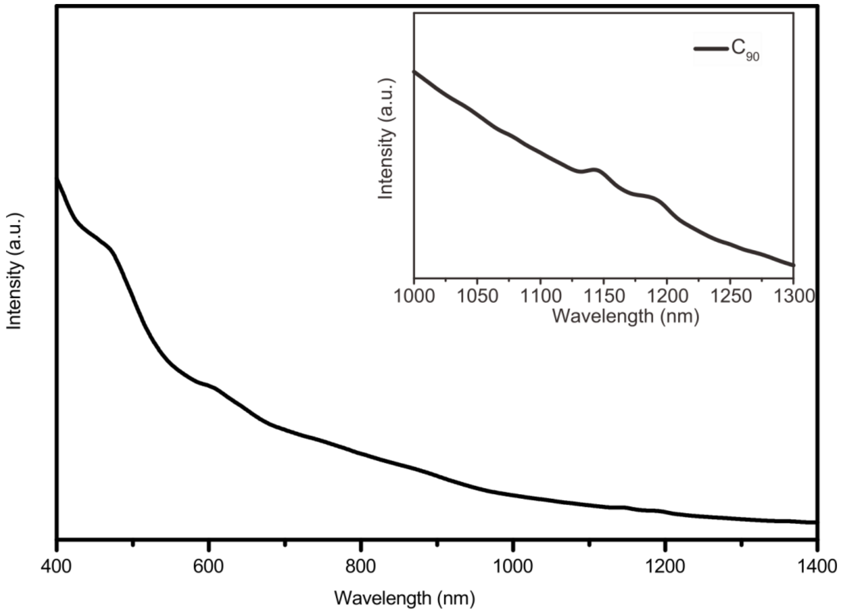

Supplementary Figure 26 | UV-Vis spectrum of C_{90} in carbon disulfide solution. Enlarged absorption spectra in the region from 1000 to 1300 nm inset.

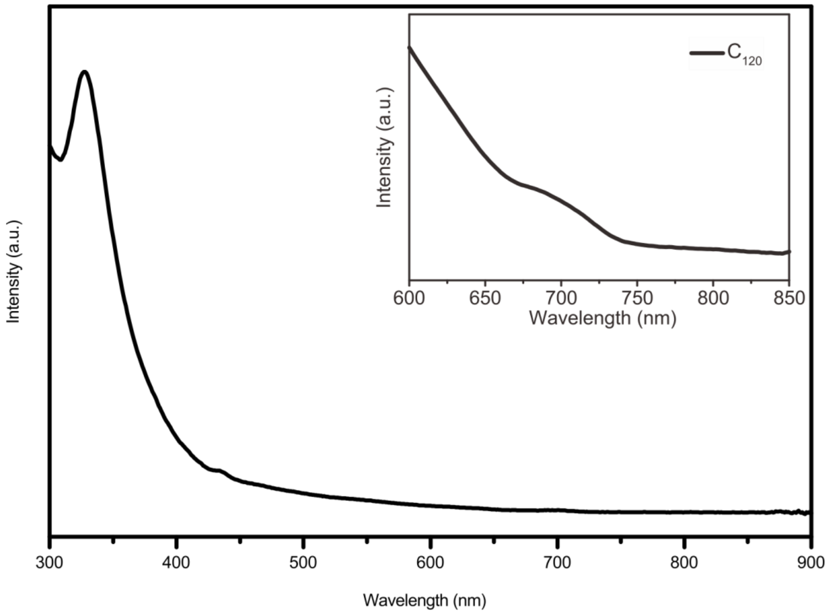

Supplementary Figure 27 | UV-Vis spectrum of C_{120} in toluene solution. Enlarged absorption spectra in the region from 600 to 850 nm inset.

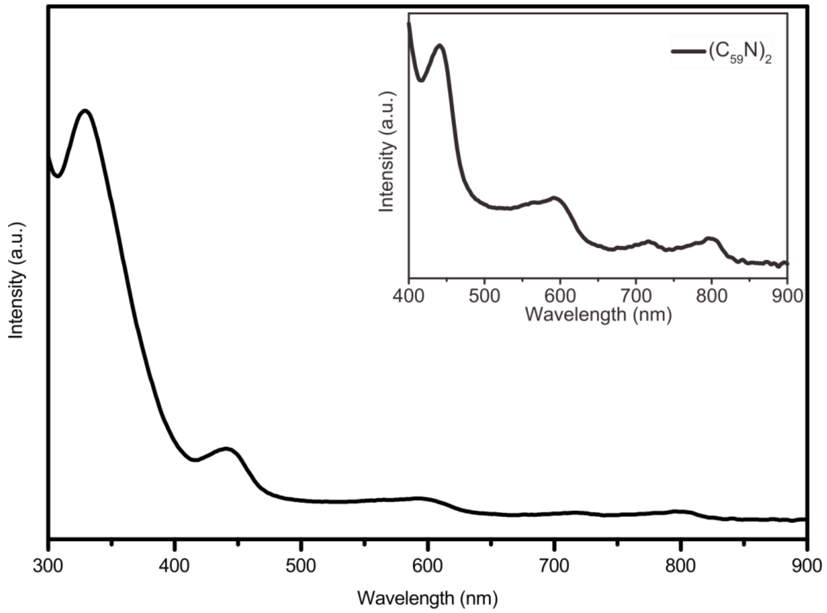

Supplementary Figure 28 | UV-Vis spectrum of $(C_{59}N)_2$ in toluene solution. Enlarged absorption spectra in the region from 400 to 900 nm inset.

Supplementary Table 1. Energy gap of the fullerenes.

Fullerene	C_{60}	C_{70}	C_{76}	C_{90}	$C_{50}H_{10}$	C_{120}	$(C_{59}N)_2$
E_g(eV)	1.93	1.87	1.36	0.98	2.51	1.67	1.49

E_g estimated from the UV-Vis spectrum onset of the absorption edge, according to an empirical formulae E_g= 1240/λ_{abs}.

Supplementary Note 3.

Equations utilized in the calculation:

The transmission spectra probability $T(E)$, probability P_i, electrical conductance G, and the average conductance $G(E_F)$ based on total energy E_i can be written as follows:

$$T(E) = Tr\left(\Gamma_L(E)G(E)\Gamma_R(E)G^{\dagger}(E)\right) \quad (1)$$

$$P_i = \frac{1}{A}e^{-\frac{E_i}{k_B T}} \quad (2)$$

$$A = \sum_{i=1}^{N} e^{-\frac{E_i}{k_B T}} \quad (3)$$

$$G_i = G_0 L_0 \quad (4)$$

$$G(E_F) = \sum_{i=1}^{N} G_i(E_F)P_i \quad (5)$$

$$L_n = \int_{-\infty}^{+\infty} dE(E - E_F)^n T(E)\left(-\frac{df(E)}{dE}\right) \quad (6)$$

Here $G_0=2e^2/h$ is the conductance quantum; h is the Planck's constant; e is the charge of a proton; $f(E) = \left(1 + \exp\left(\frac{E-E_F}{k_B T}\right)\right)^{-1}$, E_F is the Fermi energy.

C_{60}

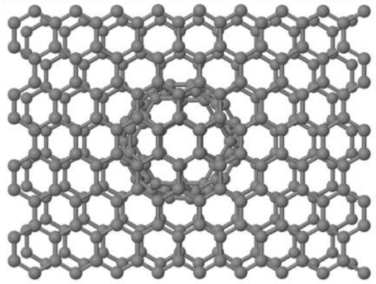

hexagon_f/hollow_g

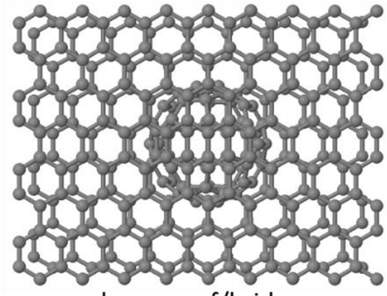

hexagon_f/bridge_g

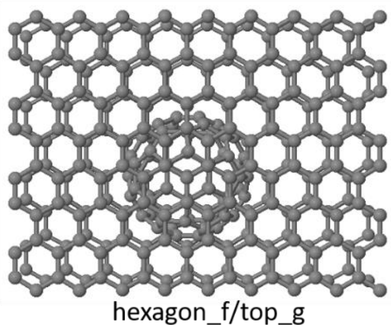

hexagon_f/top_g

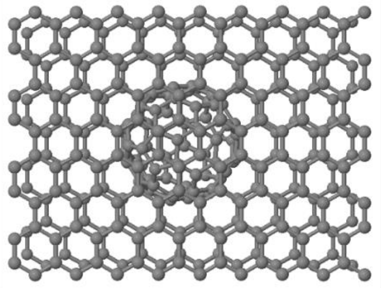

apex_f/hollow_g: rotation1

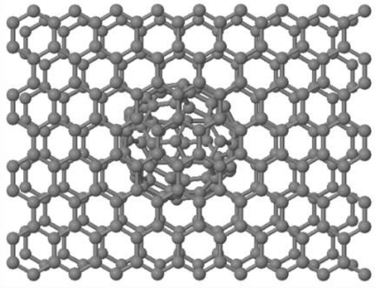

apex_f/hollow_g: rotation2

C_{70}

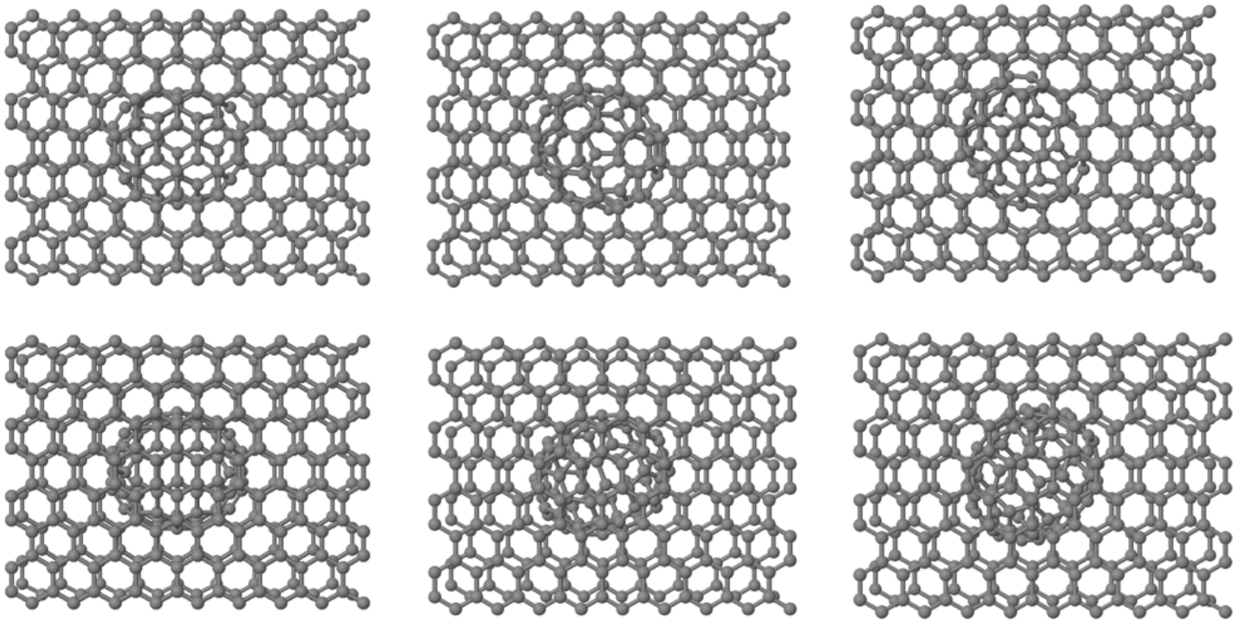

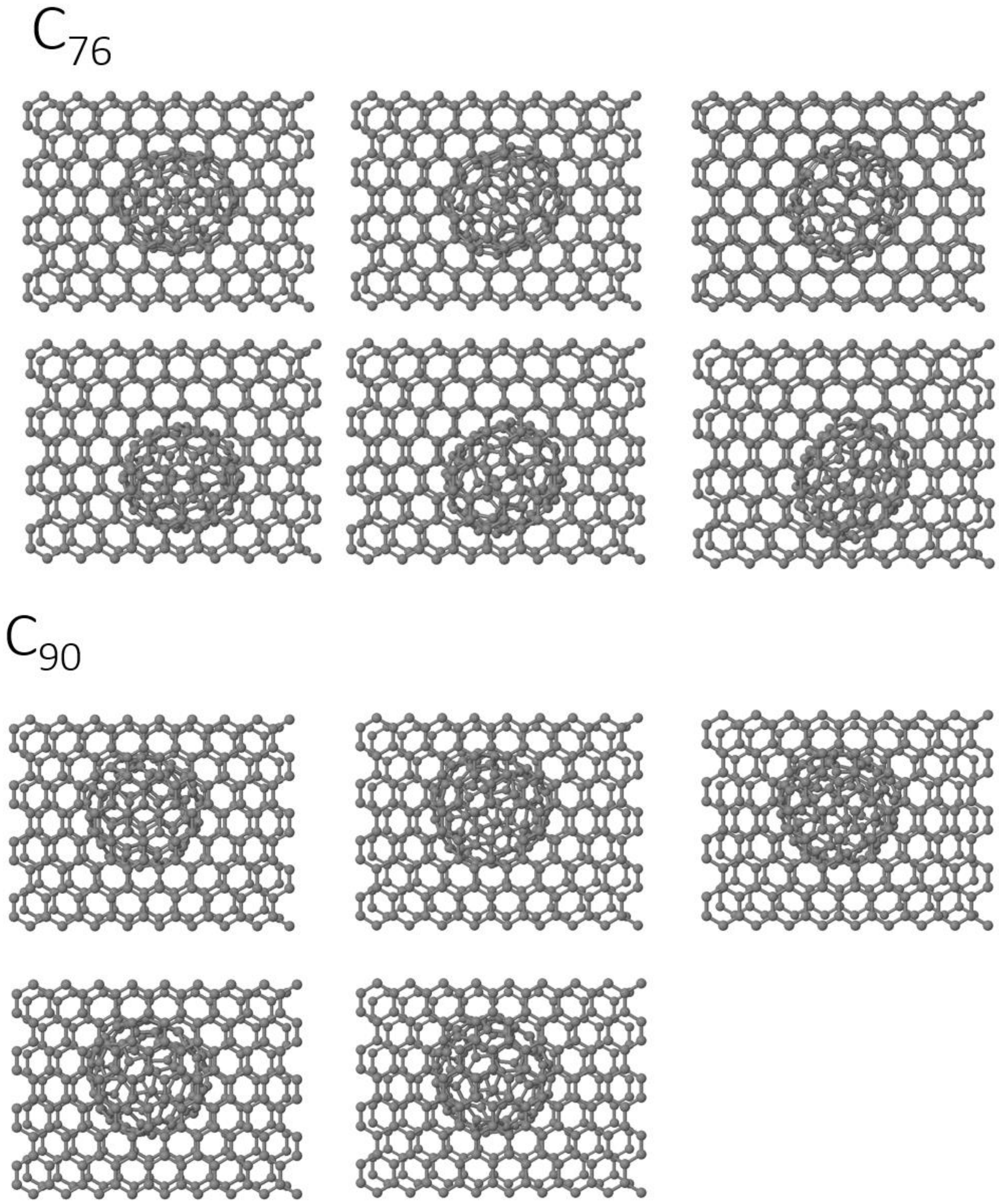

Supplementary Figure 29 | Schematic of all the configurations of the various orientations and locations of the fullerenes on the electrodes.

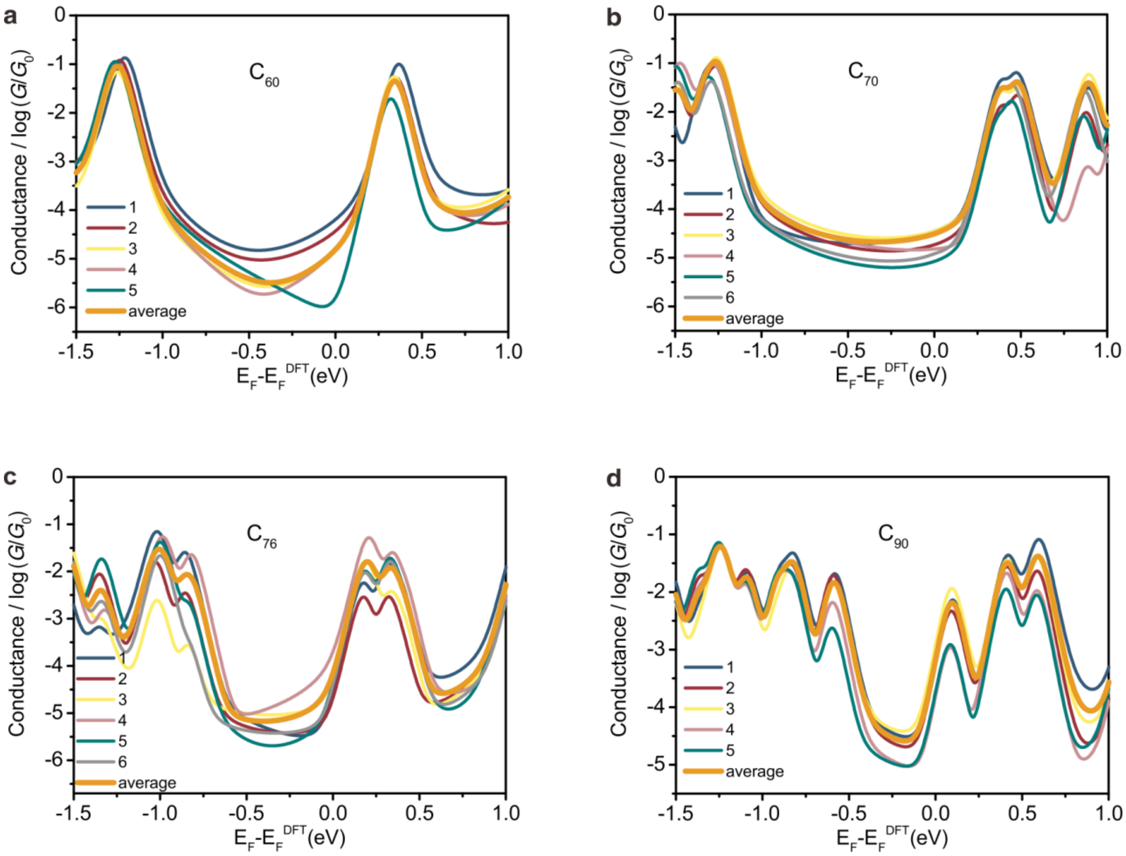

Supplementary Figure 30 | Conductance spectra in zero bias limitation as a function of Fermi levels. a, The thin curves show the conductance spectra of different configurations. The thicker orange curve is the average over these thin curves, weighted by their Boltzmann distribution. Different configurations mean different orientations and different locations of these fullerenes relative to the hexagonal lattice of graphene. **b, c, d,** have the same case with **a.**

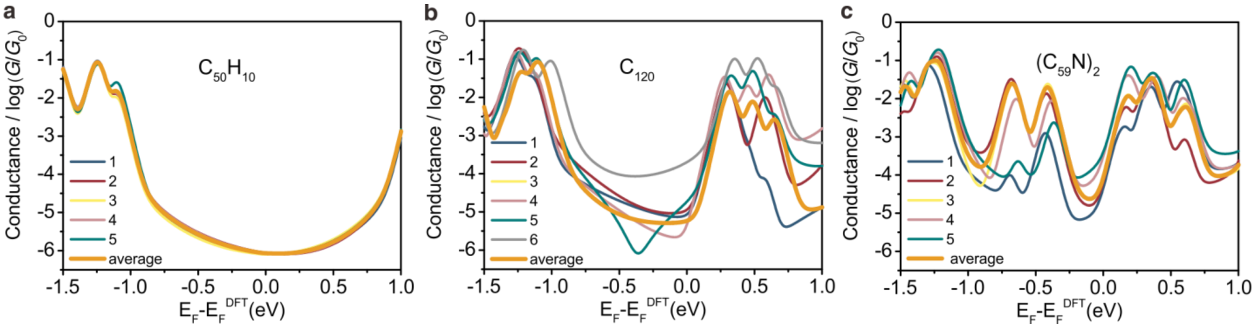

Supplementary Figure 31 | Conductance spectra in zero bias limitation as a function of Fermi levels. a, The thin curves (1, 2, 3, 4, 5) show the conductance spectra of different configurations when $C_{50}H_{10}$ is sandwiched between graphene electrodes. The thicker orange is the average over these thin curves, weighted by their Boltzmann distribution. Different configurations mean different orientations and different locations of the fullerenes relative to the hexagonal lattice of graphene. **b, c,** correspond to the molecule C_{120}, $(C_{59}N)_2$ respectively.

Supplementary Table 2. Wave functions of the frontier orbitals. The yellow atoms present carbons and the two light blue atoms in the vicinity of the connecting single bond stand for nitrogen.

HOMO<br>−4.42 eV	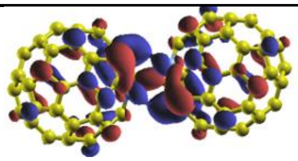	LUMO<br>−3.87 eV	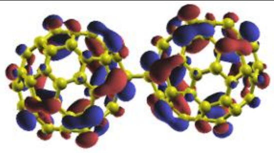
HOMO-1<br>−4.68 eV	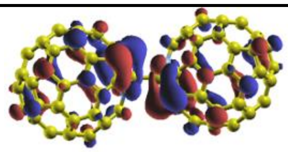	LUMO+1<br>−3.83 eV	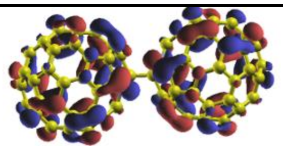
HOMO-2<br>−5.25 eV	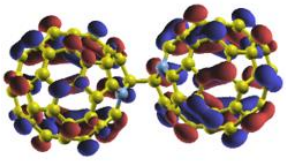	LUMO+2<br>−3.69 eV	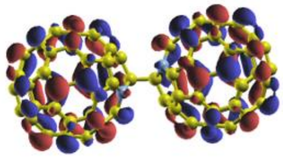
HOMO-3<br>−5.26 eV	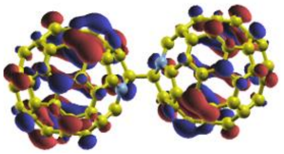	LUMO+3<br>−3.64 eV	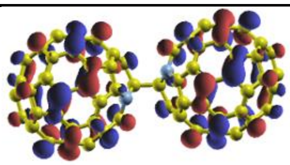

Supplementary Discussion.

The higher conductance of C_{70} is partly due to the higher coupling of the C_{70} to graphene and due to the greater density of C_{70} molecular orbital energies near the HOMO and LUMO. The greater coupling for C_{70} is illustrated by the plots below for one particular orientation of the closest fullerene hexagon relative to the graphene. This shows, for example, that the HOMO-mediated resonance of C_{70} is wider than that of C_{60}, as indicated by the red and blue arrows. This difference originates from the stronger coupling between graphene and the C_{70} molecule. Furthermore, as shown by the density of states of the gas phase C_{60} and C_{70} in Supplementary Figure 32, there are more molecular orbital energies below the HOMO (−2 eV ~ −1 eV) and more states located near

the LUMO (0 ~ 0.5 eV) for C_{70} (lower panel in d) compared to C_{60} (upper panel in d). These extra eigenstates contribute to the transmission coefficient even within the HOMO-LUMO gap and lead to an increase in the conductance of C_{70}, compared to C_{60}.

Configurations corresponding to the longer axis oriented perpendicular to the surface (Supplementary Figure 33) have a lower conductance and lower binding energy than configurations with the longer axis parallel to the surface.

Supplementary Figure 34a below shows examples of conductance versus Fermi energy for different orientations of the molecule on the surface, each with the long axis parallel to the surface. Fig. S34b shows the same curves, but with additional examples obtained when the long axis is oriented perpendicular to the surface. When E_F is close to the DFT-predicted Fermi energy E_F^{DFT} (ie when $E_F - E_F^{DFT} = 0$) the spread of conductance values is barely increased by the inclusion of the less-probable additional examples. Furthermore, since their lower binding energy renders them less probable than configurations with the long axis parallel to the surface, they barely affect the Boltzmann-weighted average conductance, shown by the blue curves in Supplementary Figure 34a, b.

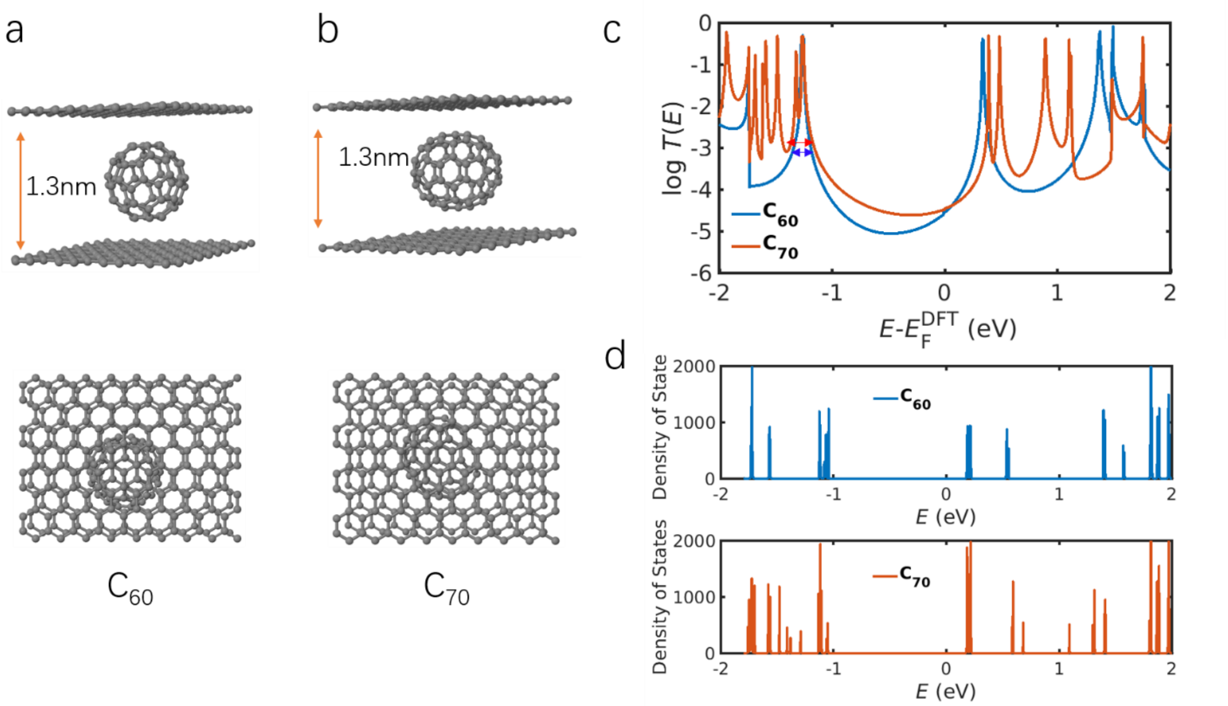

Supplementary Figure 32 | The comparison of the transport properties between C_{60} and C_{70}. **a, b,** The configurations of C_{60} and C_{70} junction with similar contact geometry. Lateral view (upper panel) and top view (lower panel). **c,** The corresponding transmission functions. **d,** The density of states of the two gas phase molecules.

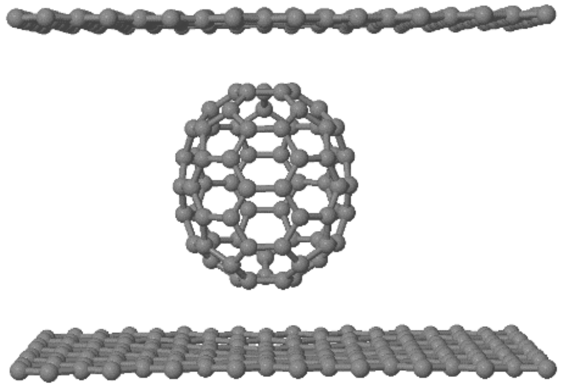

Supplementary Figure 33 | A configuration of C_{70} with the long axis perpendicular to the surface.

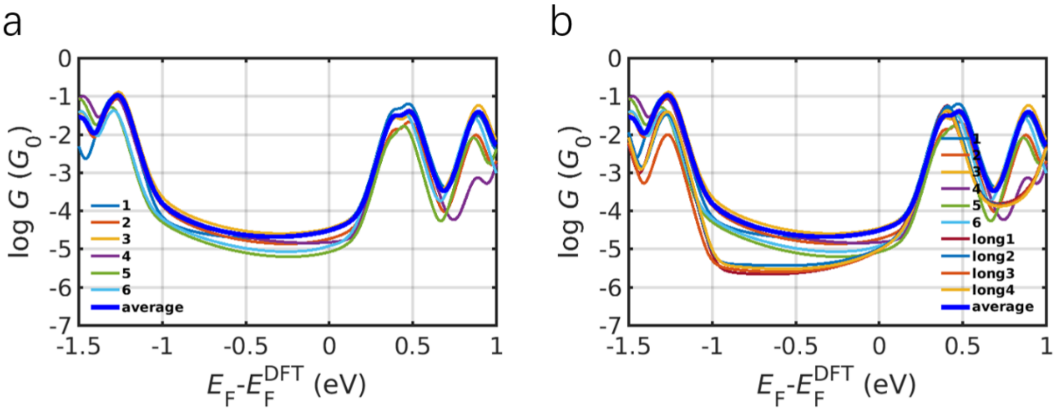

Supplementary Figure 34 | Conductance curves of different configurations of C_{70}. a, Conductance curves for 6 configurations in which the long axis is parallel to the surface. The blue line shows the Boltzmann average of these 6 curves. **b,** The conductance curves of fig. a, but with 4 more curves added, corresponding to configurations in which the long axis is perpendicular to the surface. The blue line shows the Boltzmann average of these 10 curves.
